# Silver‐enhanced Photoresponsive g‐C_3_N_4_/Ag Janus Microrobots With Negative Photogravitaxis Efficient Antibiotic Degradation

**DOI:** 10.1002/smll.202512272

**Published:** 2026-03-11

**Authors:** Yunhuan Yuan, Vinicius Tadeu Santana, Stanisław Wacławek, Michal Mazur, Martin Pumera

**Affiliations:** ^1^ Future Energy and Innovation Laboratory Central European Institute of Technology Brno University of Technology Brno Czech Republic; ^2^ Central European Institute of Technology Brno University of Technology Brno Czech Republic; ^3^ Institute For Nanomaterials Advanced Technology and Innovation Technical University of Liberec Liberec Czech Republic; ^4^ Department of Physical and Macromolecular Chemistry Faculty of Science Charles University Prague Czech Republic; ^5^ Department of Medical Research China Medical University Hospital China Medical University Taichung Taiwan; ^6^ Advanced Nanorobots & Multiscale Robotics Laboratory Faculty of Electrical Engineering and Computer Science VSB – Technical University of Ostrava Ostrava Czech Republic; ^7^ Department of Chemical and Biomolecular Engineering Yonsei University Seoul South Korea

**Keywords:** degradation mechanism, photocatalytic micromotors, ROS, three‐dimensional motion, water remediation

## Abstract

Water pollution remains a serious global challenge; hence, the efficient, low‐cost, and environmentally friendly removal of pollutants from water has become a research hotspot. Photocatalytic microrobots can achieve autonomous movement and generate reactive oxygen species (ROS), presenting a great opportunity for water purification. Despite the excellent properties of g‐C_3_N_4_, such as high photocatalytic activity, high surface area, tunable bandgap, and low cost, only a few studies have focused on g‐C_3_N_4_‐based microrobots. Herein, we developed g‐C_3_N_4_/Ag Janus microrobots that display negative photogravitaxis and efficient photocatalytic activity. Silver modification promotes the generation and separation of charge carriers while suppressing their recombination, enabling the microrobots to move upward. This three‐dimensional movement enhances mass transfer between pollutants and microrobots. We utilized the microrobots to degrade tetracycline with an efficiency of 88%, which is attributed to ROS generation and light‐driven propulsion. This work not only demonstrates negative photogravitaxis in g‐C_3_N_4_/Ag‐based microrobots but also reveals their motion‐enhanced photocatalytic mechanism for antibiotic degradation. These findings are expected to significantly advance the development of microrobotic systems for water purification and broader environmental remediation applications.

## Graphical abstract

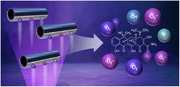

## 1 Introduction

Microrobots are micro devices that convert various forms of energy into mechanical energy to achieve autonomous movement [1, 2]. These devices have attracted increasing attention in different fields, such as environmental remediation, biomedicine applications, and sensing [3−5]. Currently, microrobot propulsion methods include chemical taxis, light taxis, magnetic and electric fields, and ultrasound [6]. Light is one of the most commonly used external stimuli due to its ability to offer precise, non‐contact control, rapid response, and a simplified system design. Photocatalytic microrobots, which can transform chemical energy and light into mechanical motion and generate reactive oxygen species (ROS), have become one of the most widely used types of light‐driven microrobots [1, 7]. Among the various developed schemes, Janus microrobots became popular [8, 9]. The most common photocatalytic materials used to fabricate Janus microrobots include titanium dioxide (TiO_2_), zinc oxide (ZnO), iron oxide (Fe_2_O_3_), and silicon dioxide (SiO_2_). These materials are often combined with a noble metal such as platinum (Pt) or gold (Au) to form Janus particles [10−13]. Previous studies have indicated that combining photocatalytic materials with a Janus structure improves charge separation and local chemical reactions under light irradiation. The resulting asymmetric distribution of reaction products enhances the overall photocatalytic efficiency and self‐propelled motion compared with symmetric photocatalytic particles [14−16].

Among various photocatalytic materials, graphitic carbon nitride (g‐C_3_N_4_) has attracted increasing interest owing to its low cost, stability, good biocompatibility, and environmental friendliness [17, 18]. However, compared to the most widely used materials, such as TiO_2_ and ZnO, g‐C_3_N_4_‐based microrobots have been less frequently reported in the literature [19, 20]. For example, Cui et al. reported Pt/Pd@g‐C_3_N_4_@carbon sphere Janus microrobots that displayed bubble‐propelled motion with speeds of 14.5 µm s^−^^1^ in 7.5% hydrogen peroxide (H_2_O_2_) solution [21]. Another study fabricated Fe/g‐C_3_N_4_ Janus micromotors propelled by self‐electrophoresis and O_2_ bubble generation, and further employed for the removal of sulfamethoxazole [22]. To the best of our knowledge, g‐C_3_N_4_/Ag microrobots have not yet been reported. Silver, a more cost‐effective choice than gold or platinum, can form a Schottky barrier, act as an electron pool, and enhance interfacial conductivity when combined with g‐C_3_N_4_, thereby increasing the utilization of photogenerated charge carriers and suppressing their recombination [23−25]. Consequently, g‐C_3_N_4_/Ag Janus microrobots are promising candidates for light‐driven microrobots.

**FIGURE 1:**
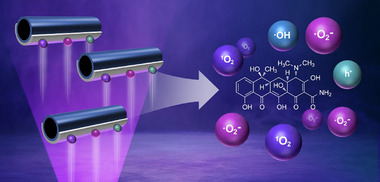
g‐C_3_N_4_/Ag Janus microrobots exhibit negative photogravitaxis and degrade tetracycline through the generation of reactive oxygen species.

In this study, we fabricated g‐C_3_N_4_/Ag Janus microrobots that can exhibit autonomous motion by photocatalytic decomposition of low‐concentration H_2_O_2_ (Figure 1). Because the introduction of Ag improves the generation and separation of charge carriers and suppresses their recombination, these microrobots can exhibit negative photogravitaxis in a 0.2% H_2_O_2_ solution. We wish to show the application of microrobots in drug remediation. Tetracycline, a commonly used broad‐spectrum antibiotic in humans and animals, can be effectively degraded by these microrobots, with a degradation efficiency of 88%. The control groups, radical scavenging experiments, and electron paramagnetic resonance (EPR) results demonstrate that the high degradation efficiency of g‐C_3_N_4_/Ag Janus microrobots results from the ROS generated by their strong photocatalytic activity and mass transfer enhanced by autonomous motion. This study contributes to the understanding of negative photogravitaxis and pollutant degradation mechanisms in g‐C_3_N_4_‐based microrobots.

## 2 Results and Discussion

**FIGURE 2:**
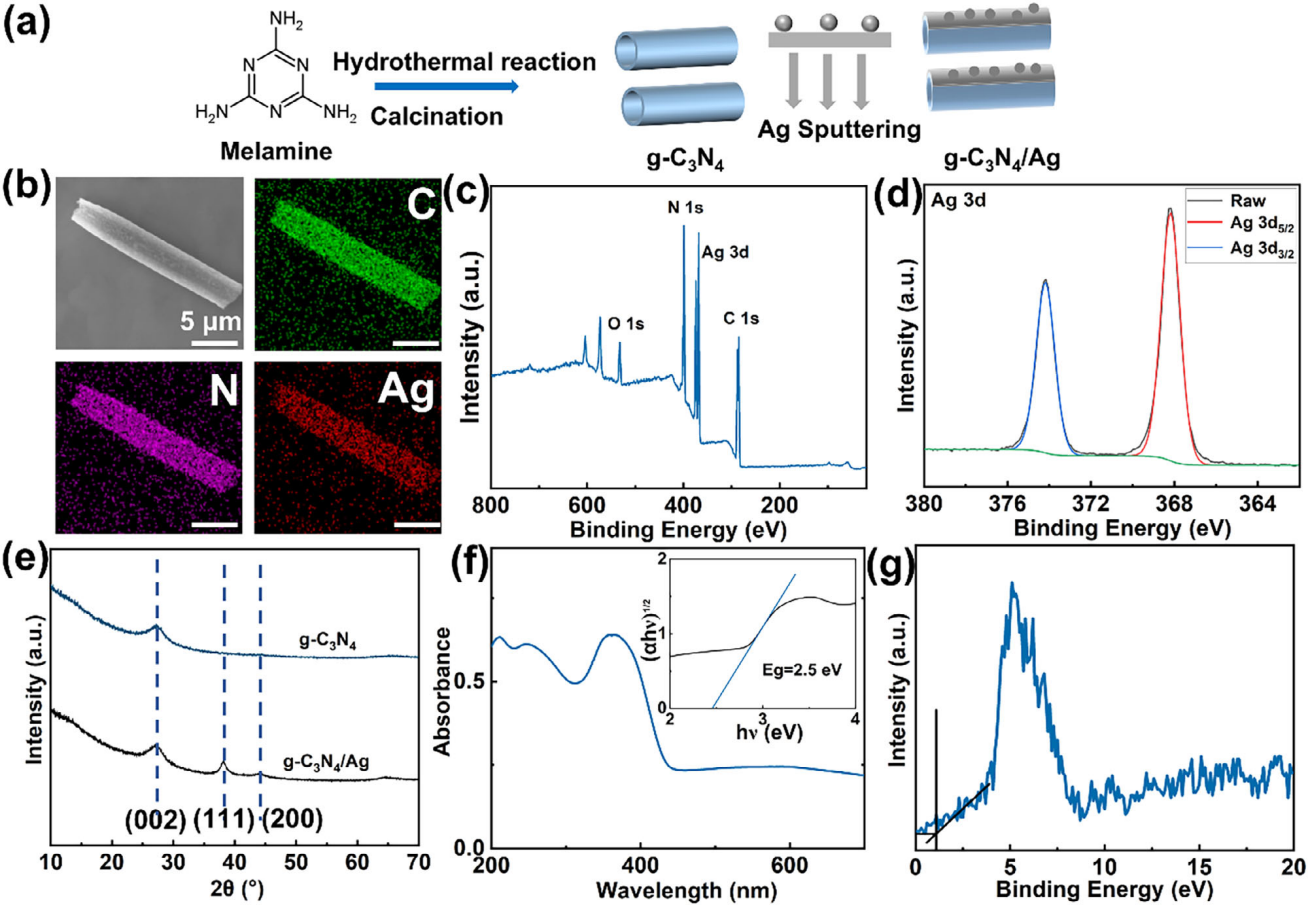
Fabrication and characterization of g‐C_3_N_4_/Ag Janus microrobots. (a) Scheme of fabrication process. (b) SEM and EDX mapping images (Scale bars in all images: 5 µm). (c) XPS survey spectrum of g‐C_3_N_4_/Ag. (d) Ag 3d XPS spectrum of g‐C_3_N_4_/Ag. (e) XRD patterns of g‐C_3_N_4_ and g‐C_3_N_4_/Ag structures. (f) UV–Vis absorption spectrum of g‐C_3_N_4_/Ag (inset: Tauc plot for evaluating the bandgap energy *E_g_*). (g) Valence band XPS spectrum of g‐C_3_N_4_/Ag.

To fabricate g‐C_3_N_4_/Ag Janus microrobots, g‐C_3_N_4_ microtubes were first prepared by combining the hydrothermal method with calcination (Figure 2a). An aqueous suspension of g‐C_3_N_4_ microtubes was then deposited onto glass substrates and allowed to dry under ambient conditions. A 15 nm‐thick Ag layer was then sputtered onto the g‐C_3_N_4_‐coated glass substrates to fabricate the g‐C_3_N_4_/Ag Janus microrobots. The scanning electron microscopy (SEM) images show their microscale tubular structures (Figure S1 and Figure 2b). Elemental analyses, including energy‐dispersive X‐ray (EDX) mapping and X‐ray photoelectron spectroscopy (XPS) survey spectra, confirm that g‐C_3_N_4_/Ag mainly contains carbon (C), nitrogen (N), and silver (Ag) (Figure 2b, c, and Figure S2). For the C 1s spectrum, four peaks, located at 284.6, 285.8, 288.0, and 288.5 eV, are assigned to C─C, C─N, N─C═N, and C═O bonds, respectively (Figure S3a). The N 1s spectrum contains three main peaks, located at 398.5, 399.4, and 400.7 eV, which are attributed to C─N═C, N─(C)_3_, and C─N─H, respectively (Figure S3b). Moreover, the small peak at 404.1 eV can be assigned to the localized positive charge in heterocyclic structures [26−28]. The Ag 3d spectrum exhibits two characteristic peaks at 368.2 eV (Ag 3d_5_/_2_) and 374.2 eV (Ag 3d_3_/_2_), with a spin–orbit splitting of 6.0 eV, corresponding to metallic silver (Ag^0^) (Figure 2d) [24, 29]. The X‐ray diffraction (XRD) pattern of g‐C_3_N_4_ displays a typical peak at 27.2° (002), which is attributed to interlayer stacking (Figure 2e) [30]. Peaks at 38.2° and 44.2° appearing in the g‐C_3_N_4_/Ag XRD spectrum can be attributed to the (111) and (200) planes of Ag, according to JCPDS 04–0783 [24]. As shown in Figure S4, Ag is uniformly distributed across the g‐C_3_N_4_ surface in the form of nanoparticles. To further analyze the interface between Ag nanoparticles and g‐C_3_N_4_, scanning transmission electron microscopy (STEM) was performed. As shown in Figure S5, STEM images reveal that Ag nanoparticles with an average size of about 6 nm are relatively uniformly dispersed on the surface of the g‐C_3_N_4_ microtubes. High‐resolution STEM imaging further shows clear lattice fringes of crystalline Ag nanoparticles in direct physical contact with the g‐C_3_N_4_ support, without obvious interfacial gaps. XPS analysis (Figure 2d) also confirms that Ag exists in the metallic state (Ag^0^). To confirm the Janus structure of the microrobots, two EDX samples were prepared from the same set of samples. In Sample A, the particles were directly transferred onto the SEM stub using copper tape with the uncoated side facing upward. In Sample B, the particles with one side sputtered with Ag were mechanically scraped off and transferred onto the SEM stub with the Ag‑coated side oriented upward. As expected, the EDX results showed that Sample B (5.2 wt.%) contained more Ag than Sample A (0.8 wt.%), supporting the presence of the Janus structure (Figure S6).

We utilized UV–Vis diffuse reflectance spectroscopy (DRS) to investigate the light absorption properties and bandgap (*E_g_*) of the g‐C_3_N_4_/Ag Janus microrobot. Figure 2f shows a characteristic absorption curve ranging from around 313 to 443 nm, with a peak located at about 363 nm. To determine the bandgap, the Tauc method was applied using the equation (αh*ν*)^1/^ *^n^* = A(h*ν* − *E_g_*), in which α is the absorption coefficient, h is Planck's constant, ν represents light frequency, A represents a constant, *E_g_* is the energy bandgap, and *n* depends on the electronic transition. For a direct transition, *n* is 1/2, while *n* is 2 for an indirect transition.[31−34] Generally, g‐C_3_N_4_ is regarded as an indirect semiconductor, and therefore *n* is 2 [19, 32]. The Tauc plot indicates that *E_g_* is around 2.5 eV. In addition, XPS valence band analysis was employed to analyze the valence band (VB), which is centered at +1.1 eV (Figure 2g) [34]. The conduction band (CB) is calculated to be −1.4 eV, following the formula *E_VB_* = *E_CB_* + *E_g_* [34].

**FIGURE 3:**
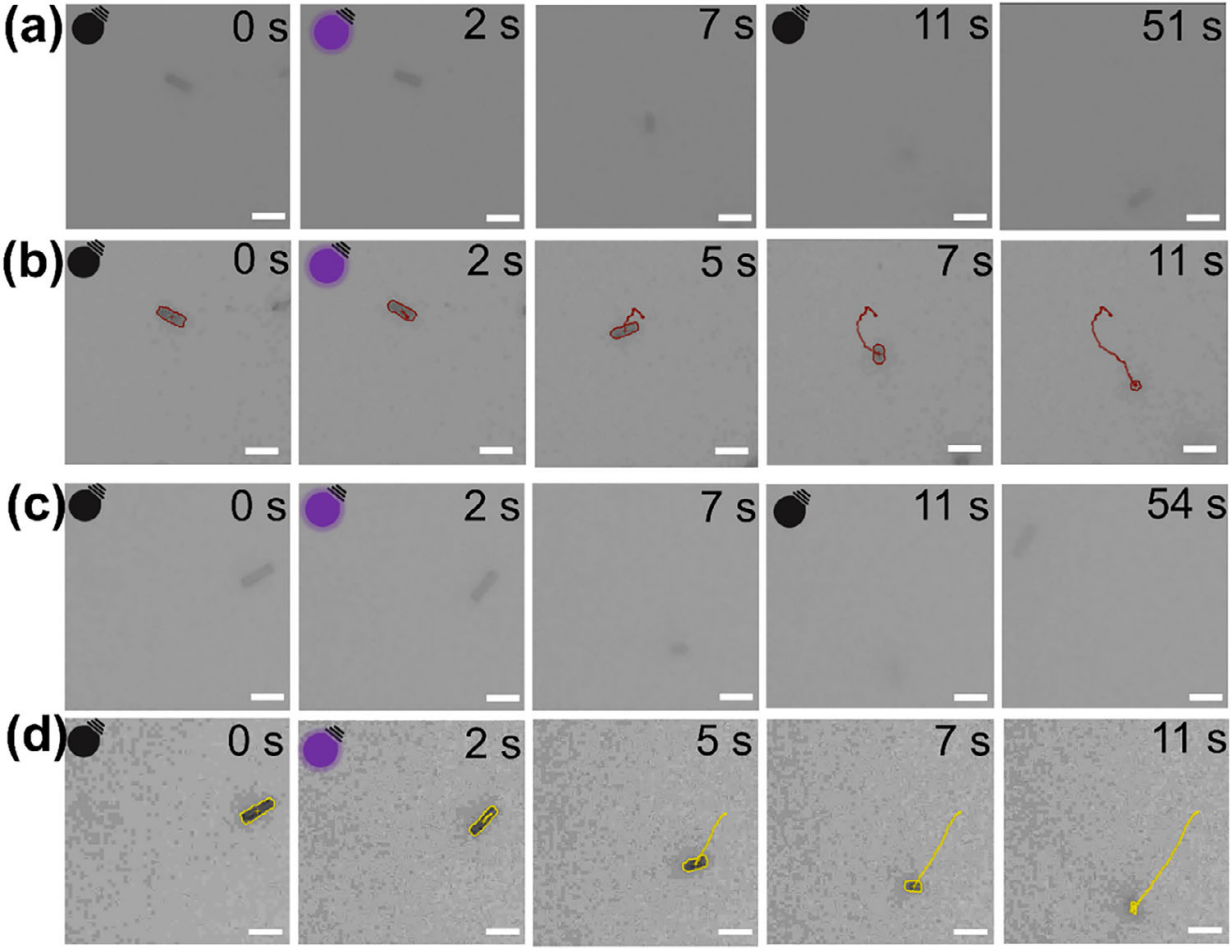
Motion behavior of g‐C_3_N_4_/Ag Janus microrobots under alternating dark and UV light conditions. (a) Representative micrographs showing typical motion in 1 wt.% H_2_O_2_ solution (dark: 0–2 s, 11–51 s; UV: 2–11 s). (b) Corresponding motion trajectories (dark: 0–2 s; UV: 2–11 s). (c) Representative micrographs of microrobots in 0.2 wt.% H_2_O_2_ solution (dark: 0–2 s, 11–54 s; UV: 2–11 s). (d) Corresponding motion trajectories (dark: 0–2 s; UV: 2–11 s). *Note*: The black lamp icon represents UV off, while the purple lamp icon represents UV on. The red line in (b) highlights the trajectory of the microrobot in 1 wt.% H_2_O_2_ solution, while the yellow line in (d) highlights its trajectory in 0.2 wt.% H_2_O_2_ solution. Scale bars in all images: 10 µm.

Subsequently, the motion behavior of g‐C_3_N_4_/Ag Janus microrobots was studied in water under 365 nm ultraviolet (UV) light, with typical videos of autonomous motion displayed in Videos S1 and S2. Figure 3 reveals the characteristic motion behaviors of Janus microrobots at varying H_2_O_2_ concentrations when exposed to alternating dark and UV light conditions. Under illumination in 1% H_2_O_2_ solution, the Janus microrobots moved rapidly and went out of focus, then completely moved out of the field of view after approximately 9 s, indicating efficient upward motion. After the light was switched off, the Janus microrobots began to refocus and gradually settled at the bottom of the substrate, becoming focused within about 40 s (Figure 3a,b, and Video S1). Under both dark and light conditions with 0.2% H_2_O_2_, the motion behaviors were similar to those observed with 1% H_2_O_2_ (Figure 3c,d, and Video S2). This form of movement is known as negative photogravitaxis, in which buoyancy and light‐driven forces to overcome the microrobots’ own weight, allowing them to move upward when exposed to vertical illumination from below [19, 35]. Negative photogravitaxis motion can improve pollutant removal efficiency in water because it is a three‐dimensional rather than two‐dimensional movement, which enhances fluid mixing and thereby promotes mass transfer and facilitates the further degradation of pollutants [36, 37].

**FIGURE 4:**
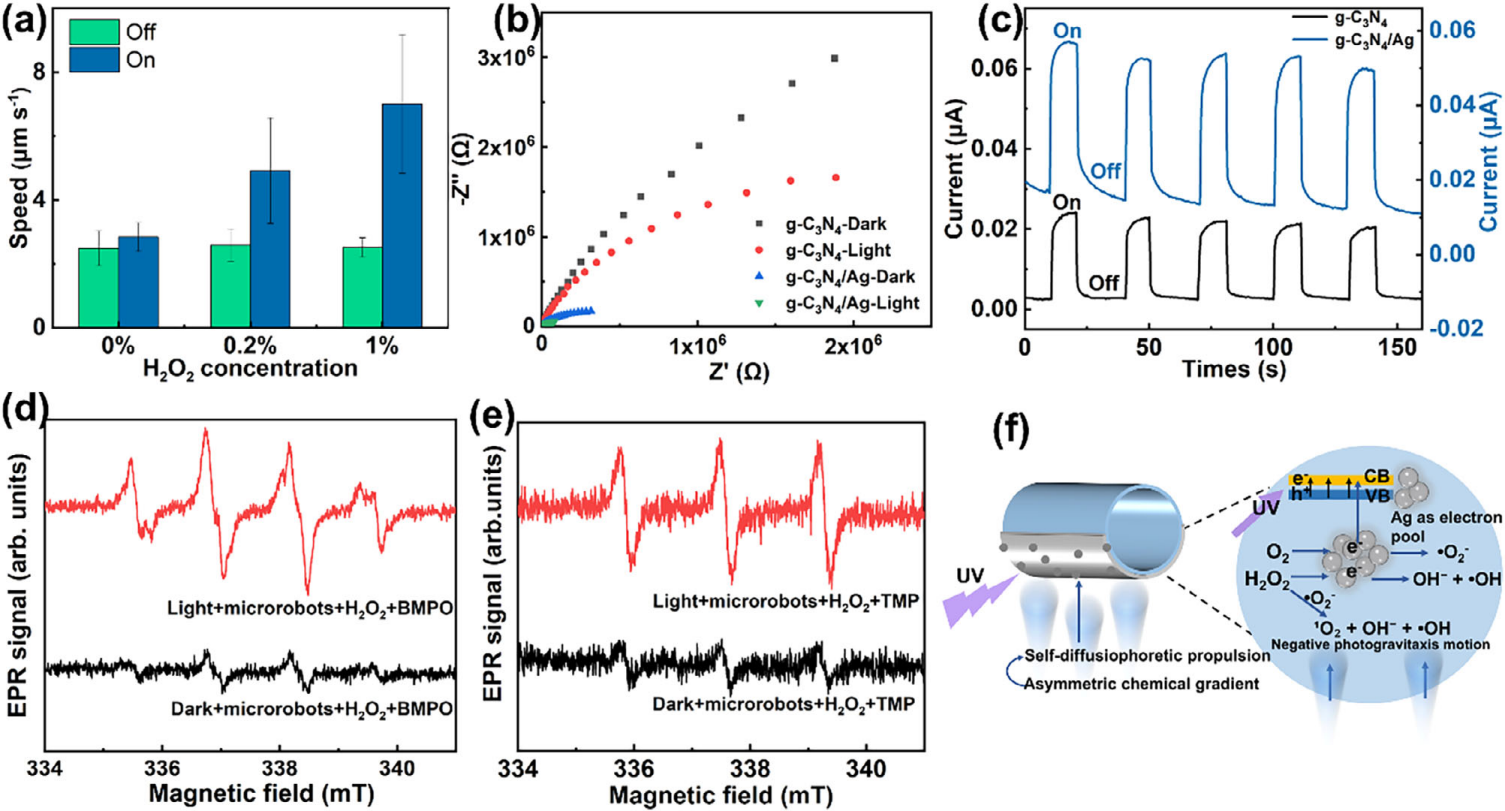
Speed of g‐C_3_N_4_/Ag Janus microrobots and investigation of their propulsion mechanism. (a) Dependence of the motion speed of microrobots on H_2_O_2_ concentration and UV light exposure. (b) EIS of g‐C_3_N_4_ and g‐C_3_N_4_/Ag under dark and light conditions. (c) Photocurrent measurements of g‐C_3_N_4_ and g‐C_3_N_4_/Ag. (d) Spin‐trapping EPR spectra of g‐C_3_N_4_/Ag using BMPO as the spin‐trap agent. (e) EPR spectra of g‐C_3_N_4_/Ag using TMP as the spin‐trap agent. (f) Illustration showing the negative photogravitaxis of g‐C_3_N_4_/Ag microrobots.

Figure 4a displays the average speed of g‐C_3_N_4_/Ag Janus microrobots in the XY plane under different conditions. The propulsion speed of the Janus microrobots changed very slightly upon light irradiation in the absence of H_2_O_2_. With 0.2% and 1% H_2_O_2_, the speeds under illumination were 1.9 and 2.8 times that under dark conditions, corresponding to 4.9 and 7 µm/s, respectively. According to previous reports, at 1% H_2_O_2_, the speed of g‐C_3_N_4_ microrobots was only 2.1 times that under dark conditions [19], indicating that silver modification facilitates the propulsion of the microrobots. In addition, in 0.2% H_2_O_2_ solution under illumination, the g‐C_3_N_4_ microrobots failed to move out of the field of view within 20 s (Video S3 and Figure S7), suggesting that the g‐C_3_N_4_/Ag Janus microrobots exhibited enhanced motion performance compared with g‐C_3_N_4_ microrobots reported previously [19].

In order to further investigate the propulsion mechanism of g‐C_3_N_4_/Ag Janus microrobots, we performed several experiments and analyzed the data, combining the experimental findings with the intrinsic properties of g‐C_3_N_4_. Given that g‐C_3_N_4_ is widely recognized as an *n*‐type semiconductor, it can be photoexcited to produce electrons and holes. In the g‐C_3_N_4_/Ag system, the work function of Ag is positioned between the conduction and valence bands of g‐C_3_N_4_, thereby promoting Schottky barrier formation at the metal–semiconductor interface (Figure S8) [19, 23, 38]. The SEM and STEM images reveal that the g‐C_3_N_4_ microstructures are uniformly decorated with Ag nanoparticles (Figures S4 and S5), which is beneficial for the formation of Schottky barriers at the interface [33, 39]. The potential well at the interface can capture electrons, facilitating electron–hole separation while suppressing carrier recombination. Under light illumination, electron–hole pairs are formed and subsequently separated [33]. Electrons excited into the conduction band of g‐C_3_N_4_ are then transferred to Ag across the Schottky barrier. The charge transport behavior was characterized using electrochemical impedance spectroscopy (EIS). Generally, the charge transfer resistance is determined by the radius of the semicircular region in the Nyquist plot, in which a smaller radius demonstrates more efficient charge transfer [39, 40]. As demonstrated in Figures 4b, g‐C_3_N_4_ exhibits a large semicircle in the dark, indicating poor charge transfer. Under light illumination, the semicircle radius decreases but remains relatively large, suggesting only limited improvement in charge transfer. In contrast, g‐C_3_N_4_/Ag displays a significantly smaller semicircle radius even under dark conditions, demonstrating lower charge transfer resistance compared to g‐C_3_N_4_. This improvement can be ascribed to the Schottky barrier formed at the g‐C_3_N_4_/Ag interface, where Ag acts as an electron pool and enhances interfacial conductivity. Under light illumination, g‐C_3_N_4_/Ag exhibits the lowest charge transfer resistance, further confirming the efficient generation and transport of photogenerated charge carriers. Photocurrent measurements were employed to further evaluate the photoinduced charge separation and transport behavior (Figure 4c) [39, 40]. In the dark, the photocurrent densities of g‐C_3_N_4_ and g‐C_3_N_4_/Ag were about 0.003 and 0.015 µA, respectively. Under light illumination, the photocurrent of g‐C_3_N_4_ increased to around 0.023 µA, while that of g‐C_3_N_4_/Ag rapidly rose to about 0.054 µA. These results further demonstrate that g‐C_3_N_4_/Ag exhibits higher efficiency in the generation and separation of photogenerated charge carriers than g‐C_3_N_4_, which can be attributed to the formation of a Schottky barrier and the enhanced interfacial conductivity provided by Ag. Moreover, the photocurrent displayed excellent photostability and reproducibility under repeated light on/off cycles. These experimental results illustrate that the introduction of Ag promotes the generation and separation of photogenerated charge carriers, suppresses their recombination, and improves overall charge transport behavior, thereby increasing the photocatalytic activity of materials. Similar EIS and photocurrent measurements have been used to demonstrate enhanced charge separation and electron transfer in photocatalytic systems [41, 42].

g‐C_3_N_4_/Ag photocatalytically decomposes H_2_O_2_ to generate chemical species such as superoxide radicals (•O_2_^−^), hydroxyl radicals (•OH), and singlet oxygen (^1^O_2_). To confirm these species, electron paramagnetic resonance (EPR) characterization was performed. The detection of •O_2_^−^ and •OH radicals was carried out using BMPO (5‐tert‐butoxycarbonyl‐5‐methyl‐1‐pyrroline‐N‐oxide), whereas TMP (2,2,6,6‐tetramethyl‐4‐piperidinol) was selectively used for trapping ^1^O_2_. Compared to the dark condition, the EPR signal of the BMPO adducts exhibited a sharp increase under light irradiation (Figure 4d), demonstrating excellent photocatalytic activity. Then, the illuminated spectrum was simulated to identify the radical adducts through the open‐source MATLAB toolbox EasySpin, which is commonly used to simulate and fit EPR spectra [43]. Figure 6b presents the experimentally obtained, simulated, and fitted EPR spectra. The results demonstrate that two spin adducts were formed, including BMPO– •O_2_^−^ and BMPO– •OH, each of which has two conformers [44, 45]. TMP, an EPR‐silent compound, can react with ^1^O_2_ to produce 4‐hydroxy‐2,2,6,6‐tetramethylpiperidine 1‐oxyl (TEMPOL), an EPR‐active species. Figure 4e displays a strong signal under 2 min light irradiation, suggesting that more ^1^O_2_ was generated. Under illumination, g‐C_3_N_4_/Ag Janus microrobots generate more chemical species, such as •O_2_^−^, •OH, and ^1^O_2_, whose uneven distribution drives diffusiophoretic propulsion, resulting in a marked increase in microrobot speed (Figure 4f) [19, 35, 46]. Similar reactive oxygen species (•OH and •O_2_^−^) have been detected and shown to play important roles in light‐driven photocatalytic systems [47, 48].

As g‐C_3_N_4_/Ag microrobots exhibit negative photogravitaxis and excellent photocatalytic properties, they show great potential for efficient pollutant degradation. Due to its broad‐spectrum antibacterial activity, tetracycline is extensively applied in human medicine, agriculture, and veterinary medicine. However, residual tetracycline has been frequently detected in its active form in wastewater, drinking water, groundwater, and sediment, posing potential risks to human health, impairing the growth of aquatic organisms, and disrupting ecological balance [49]. Thus, developing an effective, low‐cost, and environmentally friendly strategy for tetracycline degradation is of great importance. In this study, g‐C_3_N_4_/Ag microrobots enhanced for photocatalytic activity were utilized for the degradation of tetracycline.

**FIGURE 5:**
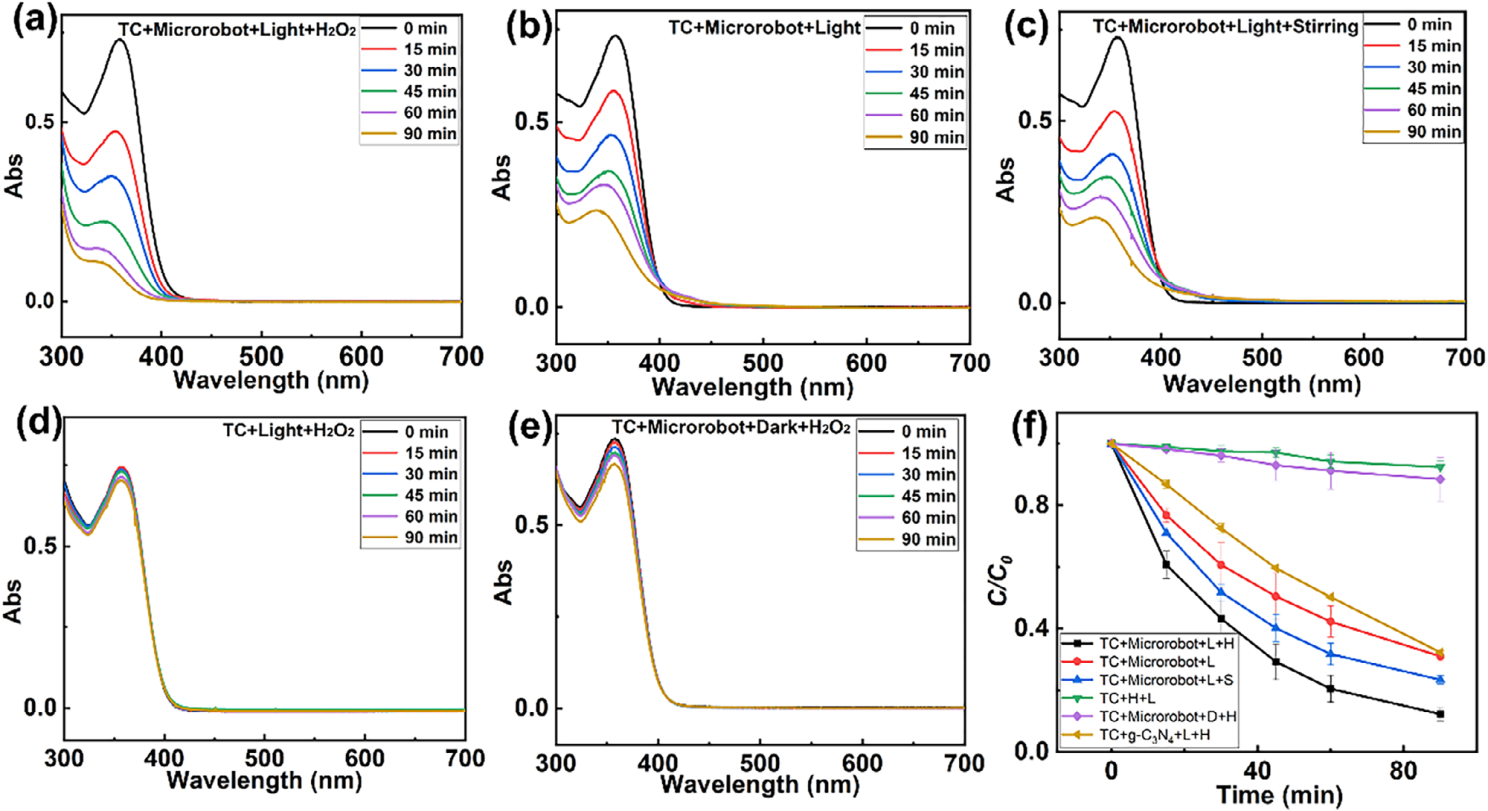
UV–Vis spectra and corresponding degradation curves of tetracycline under various experimental conditions: (a) light irradiation with microrobots and H_2_O_2_, (b) light irradiation with microrobots only, (c) light irradiation with microrobots and stirring, (d) light irradiation with H_2_O_2_ only, and (e) dark conditions with microrobots and H_2_O_2_. (f) Photocatalytic degradation curves of tetracycline over time under various conditions, including the presence or absence of microrobots, light irradiation (L), stirring (S), and H_2_O_2_ (H) (*N* = 3).

Figure 5a shows a gradual reduction in the absorption intensity of tetracycline when exposed to light in the presence of H_2_O_2_ and microrobots. This time‐dependent reduction in absorbance corresponds to a degradation efficiency of 88% within 90 min (Figure 5a, f). However, the degradation efficiency is only 69% in the absence of H_2_O_2_ (Figure 5b, f). To further investigate the contribution of the intrinsic photocatalytic activity, a non‐Janus g‐C_3_N_4_/Ag composite with a similar Ag loading (1.8%, compared to 2.3% in the Janus microrobots) was synthesized and used as a control material (Figure S9). Under the same reaction conditions (light irradiation and 0.2% H_2_O_2_), the non‐Janus composite achieved a tetracycline degradation efficiency of 77% (Figure S10), while the Janus microrobots reached 88%. Besides, to explore the effect of mass transfer on degradation, experiments with slow stirring were performed to simulate enhanced fluid motion. Under slow stirring condition, the efficiency increased to 77% (Figure 5c, f), indicating that improved mass transfer contributes to higher degradation efficiency. Taken together, these findings suggest that the photocatalytic activity of g‐C_3_N_4_/Ag plays the primary role in tetracycline degradation, while the self‐propulsion of the microrobots enhances mass transfer, leading to an additional degradation effect. Another control experiment (Figure 5d, f) shows that in the presence of H_2_O_2_ alone, the degradation efficiency is only 8%, which can be attributed to the limited ROS production under light irradiation without microrobots. These results suggest that the addition of H_2_O_2_ improves the degradation efficiency by both enabling microrobot propulsion, which enhances fluid mixing and thereby facilitates mass transfer, and increasing ROS generation under light irradiation. Under dark conditions, the degradation efficiency is 12% (Figure 5e, f), which can be attributed to the limited ROS production. Besides, Figure 5f shows that the degradation efficiency of pristine g‐C_3_N_4_ with H_2_O_2_ under light irradiation is only 68%, suggesting that Ag modification can indeed improve the degradation performance.

**FIGURE 6:**
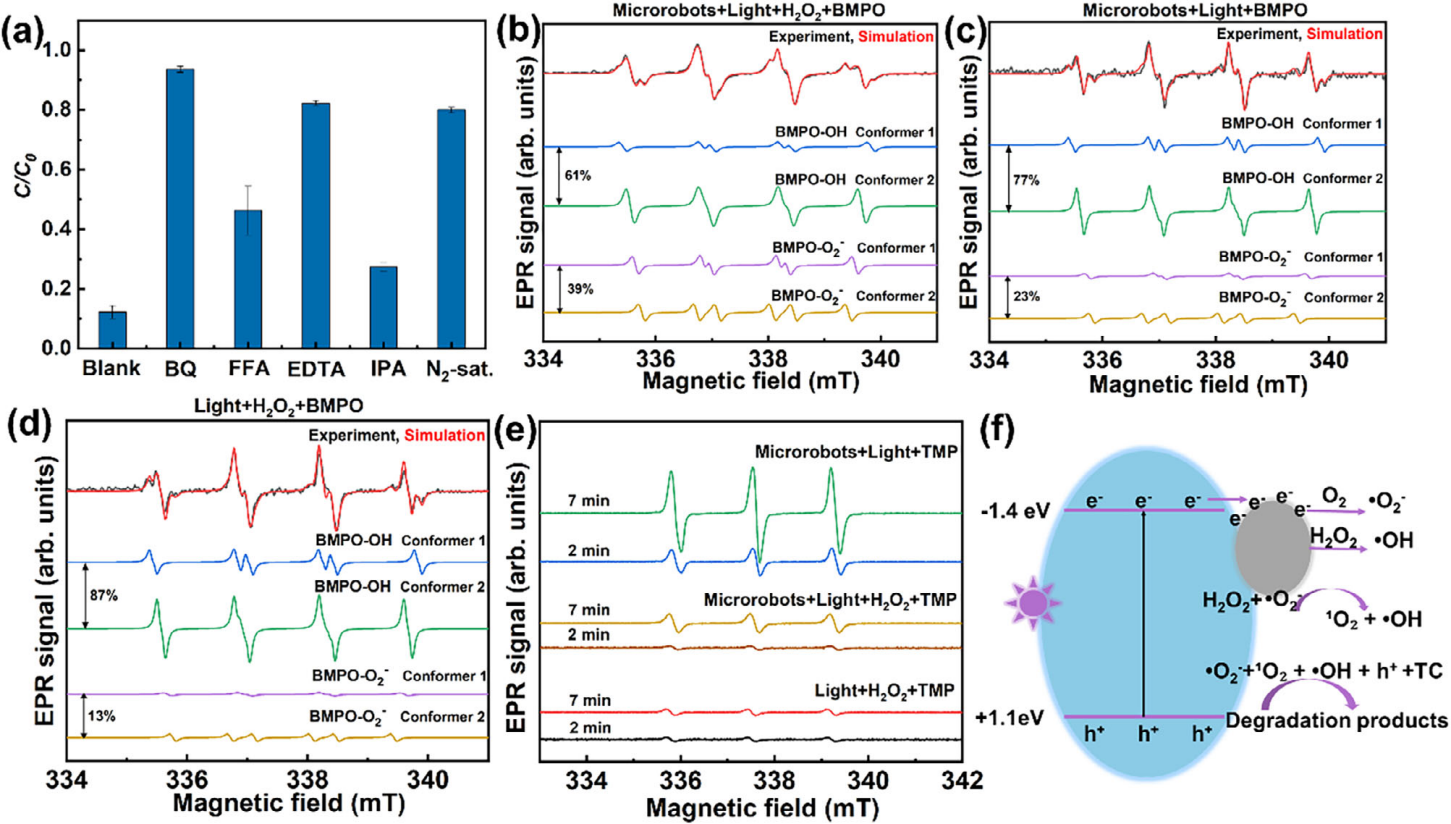
Mechanistic investigation of tetracycline degradation. (a) Degradation efficiency of tetracycline with various radical scavengers and under N_2_‐saturated conditions. (b) Simulated EPR spectra with g‐C_3_N_4_/Ag microrobots and H_2_O_2_ under light (BMPO used as the spin‐trap agent). (c) Simulated EPR spectra with g‐C_3_N_4_/Ag microrobots only under light (BMPO). (d) Simulated EPR spectra with H_2_O_2_ only under light (BMPO). (e) EPR spectra under different conditions using TMP as the spin‐trap agent. (f) Schematic illustration showing the tetracycline degradation mechanism.

Due to their high photocatalytic efficiency, tunable band structure, good stability, and environmental friendliness, g‐C_3_N_4_‐based materials have been widely applied as photocatalysts in the degradation of organic pollutants, and their underlying mechanisms have been extensively studied [50, 51]. Upon light absorption, electrons in g‐C_3_N_4_ transition from the valence band to the conduction band, leaving holes in the valence band and thereby promoting the separation of photogenerated charge carriers. Electrons and holes generated on the photocatalyst surface can interact with oxygen (O_2_), water (H_2_O), or hydrogen peroxide (H_2_O_2_) to produce reactive species. These species generated from the above processes, including superoxide radicals (•O_2_^−^), holes (h^+^), singlet oxygen (^1^O_2_), and hydroxyl radicals (•OH), can disrupt the structure of organic compounds, thereby resulting in their degradation. To determine the roles of these active species, radical scavenging experiments were conducted. Benzoquinone (BQ), furfuryl alcohol (FFA), ethylenediaminetetraacetic acid (EDTA), and isopropanol (IPA) are the corresponding scavengers for •O_2_^−^, ^1^O_2_, h^+^, and •OH, respectively [19, 52]. Due to the addition of BQ, the degradation efficiency of tetracycline dropped sharply from 88% to 6% (Figure 6a), demonstrating that •O_2_^−^ plays the most important role in the degradation. •O_2_^−^ contributes to the degradation of tetracycline through two pathways. The first is a direct interaction between •O_2_^−^ and tetracycline while the second involves a secondary reaction with H_2_O_2_ to produce ^1^O_2_ according to the reaction H_2_O_2_ + •O_2_^−^ → ^1^O_2_ + OH^−^ + •OH [53, 54, 55]. As shown in Figure 6a, when ^1^O_2_ is scavenged, the degradation efficiency decreases to 54%, confirming that ^1^O_2_ is also a crucial species in the degradation process. Moreover, when •O_2_^−^ is scavenged, the formation of ^1^O_2_ is also inhibited, leading to a significantly reduced degradation efficiency. It is noted that the degradation efficiency dropped to 18% after the addition of EDTA, which may be attributed to the formation of EDTA–Ag complexes that block active sites and hinder electron transfer [56, 57]. After the addition of IPA, the degradation efficiency remained relatively high at 73%. Thus, h^+^ and •OH are not the primary reactive species responsible for the degradation process. Moreover, the degradation efficiency significantly dropped to 20% under N_2_‐saturated conditions, indicating a key contribution of photogenerated electrons to the photocatalytic mechanism. Figures 6b–d display the simulated EPR spectra under different conditions upon light irradiation. The hyperfine coupling parameters used to simulate, identify, and quantify the relative concentration between BMPO adducts are in Table S1. The spectra demonstrate that •O_2_^−^ accounts for 39% of the BMPO adducts in the presence of g‐C_3_N_4_/Ag microrobots and H_2_O_2_, which is higher than that with g‐C_3_N_4_/Ag alone (23%) or H_2_O_2_ alone (13%). These results further confirm that •O_2_^−^ plays a significant role in the degradation process. Figure 6e shows that under light irradiation, the conversion of the EPR‐silent species TMP into the EPR‐active TEMPOL increases over time due to its reaction with ^1^O_2_. When only H_2_O_2_ is present, the ^1^O_2_ signal remains nearly unchanged. However, a slight increase is observed when both the microrobots and H_2_O_2_ are present. Notably, the signal increases significantly in the presence of microrobots alone, indicating that the microrobots can more effectively convert •O_2_^−^ into ^1^O_2_ through disproportionation [52, 53, 58]. The proposed photodegradation mechanism of the g‐C_3_N_4_/Ag microrobots is illustrated in the schematic diagram (Figure 6f). Upon light irradiation, photoexcited electrons transition from the valence band to the conduction band and subsequently transfer to Ag nanoparticles. Due to the more negative conduction band position (−1.4 eV) compared to the O_2_/•O_2_^−^ redox potential (−0.33 eV), the photogenerated electrons can efficiently reduce O_2_ to •O_2_^−^. Some of the •O_2_^−^ further react with H_2_O_2_ to generate ^1^O_2_. In addition, a portion of H_2_O_2_ can directly react with electrons, leading to the formation of •OH. Herein, Ag enhances interfacial conductivity and acts as an electron pool that captures photogenerated electrons, thereby facilitating charge separation and suppressing recombination. Finally, the reactive species, including •O_2_^−^, ^1^O_2_, h^+^, and •OH, attack tetracycline, leading to its degradation.

To investigate the degradation products, HPLC–MS analysis was performed on the tetracycline solution before and after photocatalytic treatment. The results are presented in Figure S11. As shown in Figure S11a, the total ion chromatograms (TIC) reveal multiple degradation product peaks at 3–5 min retention time in the photocatalytically treated sample, while these peaks are completely absent in the untreated control. This demonstrates that the photocatalytic process induces chemical transformation of tetracycline rather than simple adsorption or non‐specific binding to the photocatalyst surface.

To identify the degradation intermediates, extracted ion chromatograms (EICs) were obtained at specific m/z values corresponding to tetracycline transformation products (Figure S11b). The analysis revealed the presence of an oxidized tetracycline form at m/z 418.130 (TCKOx, corresponding to C_22_H_23_N_2_O_9_^+^), a dehydrated tetracycline form at m/z 416.133 (TCKdehyd, C_22_H_22_NO_9_^+^), and a hydroxylated derivative at m/z 434.166. Additionally, two unidentified transformation intermediates were detected at m/z 418.170 and 416.157, which likely possess different elemental compositions compared to the characterized oxidized and dehydrated forms. All of these intermediates were detected exclusively in the treated sample with retention times between 7–9 min, while being completely absent in the untreated solution. The detection of multiple transformation products with distinct m/z values indicates that several parallel degradation pathways occur simultaneously during photocatalysis. The appearance of these specific products provides direct evidence for degradation pathways mediated by reactive oxygen species, such as ·O_2_^−^ and ·OH. The identified intermediates are consistent with previous reports [59] showing that tetracycline undergoes, for example, dehydration reactions in the presence of photogenerated reactive species, resulting in multiple transformation intermediates with different elemental compositions.

To evaluate the environmental risk of the photocatalytic process, the concentration of dissolved Ag^+^ in the solution before and after degradation was measured using inductively coupled plasma optical emission spectrometry (ICP‐OES, Thermo Scientific iCAP PRO). The Ag^+^ concentration decreased slightly from 764 µg/L (before reaction) to 636 µg/L (after light irradiation with H_2_O_2_). These results indicate that light and H_2_O_2_ did not lead to Ag^+^ leaching under the experimental conditions. The decrease may be attributed to the photoreduction and redeposition of Ag^+^ onto the g‐C_3_N_4_/Ag surface and the adsorption of Ag^+^ onto the material [60]. Overall, the photocatalytic process does not result in additional Ag^+^ release and is unlikely to pose increased environmental risks.

The g‐C_3_N_4_/Ag Janus microrobots were further evaluated in real wastewater to assess their potential for practical application. As shown in Figure S12, the photocatalytic degradation efficiency in the real wastewater reached 82%, which is even slightly lower than that obtained in deionized water. These results indicate that the presence of background ions and organic constituents did not obviously hinder the photocatalytic performance of the microrobots. Overall, the microrobots exhibit stable and robust degradation behavior in realistic aqueous environments, highlighting their potential for practical wastewater treatment applications.

## 3 Conclusion

The g‐C_3_N_4_/Ag Janus microrobots exhibit autonomous propulsion and remarkable photocatalytic activity. They display negative photogravitaxis and move out of the microscope's focus in less than 10 s in a 0.2% H_2_O_2_ solution, indicating efficient upward movement. In addition, the microrobots degrade tetracycline with an efficiency of 88%. This performance is attributed to the introduction of silver, which enhances photocatalytic activity by promoting the generation and separation of charge carriers and suppressing their recombination. Furthermore, experimental results demonstrate that the microrobots’ motion improves mass transfer, thereby facilitating antibiotic degradation. EPR and ROS scavenger experiments further reveal that superoxide radicals play the dominant role in the degradation process. This work deepens the understanding of negative photogravitaxis and the antibiotic degradation mechanism of g‐C_3_N_4_‐based microrobots, which is expected to have a significant impact on the development of microrobot technologies for wastewater treatment and pharmaceutical pollutant removal.

## 4 Experimental Section

### 4.1 Fabrication of g‐C_3_N_4_/Ag Janus Microrobots

For preparing the Janus microrobots, g‐C_3_N_4_ microtubes were first fabricated using a hydrothermal method and calcination. A mixture of 1 g melamine, 0.4 g NaOH, and 100 mL deionized water was stirred for 5 min. After being heated at 70 °C for 20 min to form a transparent solution, the mixture was placed in a Teflon‐lined autoclave and maintained at 180 °C for 14 h in an oven. Once cooled, the resulting precipitate was collected by centrifugation, washed repeatedly using ethanol and deionized water, and subsequently dried at 50 °C to obtain a white powder. The powder was added to a crucible and subsequently treated in a tube furnace at 500 °C for 90 min under a nitrogen environment to produce g‐C_3_N_4_ microtubes. Next, the g‐C_3_N_4_ microtubes were dispersed in deionized water, ultrasonicated, and then coated onto glass substrates. After drying, a 15 nm‐thick Ag layer was deposited using sputter coating to form the g‐C_3_N_4_/Ag Janus microrobots. The non‐Janus g‐C_3_N_4_/Ag composite was synthesized using a UV photodeposition method, similar to the procedure reported in Materials Research Bulletin [61]. Specifically, 6 mg of g‐C_3_N_4_ microtubes were ultrasonically dispersed in 15 mL of deionized water. Then, 0.0084 g of AgNO_3_ was added to the dispersion, and the mixture was stirred for 4 h. The resulting suspension was centrifuged to remove the supernatant. The precipitate was redispersed in 15 mL of deionized water, stirred, and irradiated under UV light for 3 h. The resulting product was collected by centrifugation, washed three times with deionized water, and dried in a vacuum oven at 50°C to obtain the non‐Janus g‐C_3_N_4_/Ag composite.

### 4.2 Characterization

A scanning electron microscope (MIRA3 XMU), which has an EDX detector, was employed to obtain SEM images and EDX mapping. To obtain the distribution of silver, a JEOL JEM NEOARM‐200F transmission electron microscope (STEM) operating in scanning mode was used. Images were obtained using annular dark field (ADF) and annular bright field (ABF) detectors at an accelerating voltage of 200 kV. XRD patterns were recorded using a Rigaku SmartLab diffractometer. XPS analysis was performed using a KRATOS Axis Supra system. A V‐730 UV–Vis spectrophotometer was employed to measure the absorption spectra. The g‐C_3_N_4_/Ag Janus microrobots were further analyzed for their optical absorption and bandgap using UV–Vis diffuse reflectance spectroscopy (DRS). A Thermo Scientific iCAP PRO instrument was used to measure the concentration of dissolved Ag^+^ in the solution before and after the photocatalytic reaction.

### 4.3 Microrobots’ Motion Behavior

The microrobots’ motion was captured using a Nikon ECLIPSE Ts2R inverted microscope integrated with a BASLER acA1920‐155uc imaging system. For each test, 10 µL of g‐C_3_N_4_/Ag Janus microrobot suspension and 10 µL of H_2_O_2_ solution at different concentrations were mixed to achieve final concentrations of 0.2 and 1 wt.%. The 365 nm UV light‐emitting diodes (CoolLED pE‐100) acted as the light source. The microrobots’ motion videos were acquired under both dark and light conditions with a frame rate of 25 fps. Microrobot trajectories and speeds were analyzed using the NIS‐Elements Advanced Research software. Each speed dataset was acquired by analyzing 20 particles. There were no surfactants used in the experiments.

### 4.4 Electrochemical Impedance Spectroscopy (EIS) and Photocurrent Response Measurements

To prepare a uniform suspension, 0.5 mg of g‐C_3_N_4_ and g‐C_3_N_4_/Ag powders were individually added to a mixture consisting of 0.25 mL of ethanol, 0.25 mL of water, and 5 µL of Nafion (5%), followed by sonication for 5 min. Then, 5 µL of each dispersion was separately dropped onto screen‐printed carbon electrodes (SPCEs) and dried for further analysis. A conventional three‐electrode setup was employed for all electrochemical measurements using an electrochemical workstation, where 0.25 m K_2_SO_4_ acted as the electrolyte solution. In detail, the reference and counter electrodes were an Ag/AgCl electrode and a platinum wire, respectively, while the sample‐modified SPCEs worked as the working electrode. The charge transfer resistance of the samples under dark and illuminated conditions was tested using EIS over a frequency range of 100 kHz to 1 mHz. Photocurrent measurements were performed at a fixed potential of 0.6 V using a chronoamperometric technique, and photocurrent density–time curves were recorded with light on/off cycles.

### 4.5 Electron Paramagnetic Resonance (EPR) Experiments

A Magnettech X‐band EPR spectrometer equipped with a cuvette (70 µL) suitable for aqueous samples was used to perform the EPR measurements. For the detection of •O_2_^−^ and •OH, the aqueous sample contained 14 mm BMPO, 0.16 mg/mL g‐C_3_N_4_/Ag, and 0.2% H_2_O_2_. The signals under both dark and illuminated conditions were accumulated 9 times to improve the signal‐to‐noise ratio since a low modulation was used to avoid overmodulating narrow peaks from different components in the spectra. Under illumination, signal acquisition began after 2 min of light exposure. For the detection of ^1^O_2_, the sample consisted of 25 mm TMP, 0.16 mg/mL g‐C_3_N_4_/Ag, and 0.2% H_2_O_2_. In this measurement, the signal was collected without accumulation, and acquisition began after 2 and 7 min of light exposure, respectively. The light source used was the same as that employed for observing the microrobots’ motion behavior. All experiments were conducted in MilliQ water. The spectra were obtained under experimental conditions of 3.9 mW microwave power, 0.04 mT modulation amplitude, and 100 kHz frequency. Two control groups were included: one containing the spin‐trap agent (BMPO or TMP) and g‐C_3_N_4_/Ag without H_2_O_2_, and the other containing the spin‐trap agent (BMPO or TMP) and H_2_O_2_ without g‐C_3_N_4_/Ag. The experimental conditions for both groups were the same as those described above. The EPR spectra were simulated and fitted using the *garlic* function from EasySpin, an open‐source MATLAB toolbox for EPR data analysis that considers the averaging of anisotropies in the liquid phase due to molecular tumbling [43].

### 4.6 Photocatalytic Degradation of Tetracycline (TC)

A 20 mL glass vial was employed to perform the degradation experiments with g‐C_3_N_4_/Ag Janus microrobots. 1.6 mg of g‐C_3_N_4_/Ag was introduced into 16 mL of a tetracycline solution (2.5 mg/100 mL) containing 0.2% H_2_O_2_, followed by 3 min of ultrasonication to achieve a uniform suspension. Then the sample was illuminated with a 365 nm light source (P‐LAB, 8 W). At specific time intervals, 2 mL of the mixture was collected and passed through a polyvinylidene fluoride (PVDF) membrane to remove the microrobots. The degradation efficiency was determined by measuring the absorbance of the resulting solution at the characteristic wavelength of 357 nm using a UV–Vis spectrophotometer. To explore the degradation mechanism, a series of control experiments were conducted under the following conditions: (1) under light with g‐C_3_N_4_/Ag but without H_2_O_2_; (2) under light with g‐C_3_N_4_/Ag and stirring (100 rpm) but without H_2_O_2_; (3) under light with H_2_O_2_ but without g‐C_3_N_4_/Ag; and (4) with both g‐C_3_N_4_/Ag and H_2_O_2_ but in the dark. In addition, a control experiment using g‐C_3_N_4_ and H_2_O_2_ (without Ag) was performed to evaluate the specific contribution of Ag. The degradation efficiency was determined through the equation: removal efficiency (%) = (*C_0_* − *C*) / *C_0_* × 100%, where *C_0_* and *C* represent the initial and time‐dependent concentrations of tetracycline, respectively. In addition, to explore the contributions of reactive species for degradation, radical scavenging experiments were carried out by introducing 1 mmol of BQ (for •O_2_^−^), 1 mmol of FFA (for ^1^O_2_), 1 mmol of EDTA (for h^+^), and 1 mmol of IPA (for •OH) as specific quenchers.

### 4.7 HPLC–MS Analysis

The degradation intermediates of tetracycline were analyzed using a Sciex X500R mass spectrometer coupled to an ExionLC AC liquid chromatograph. A YMC Triart C18 column (100 mm × 2.1 mm, 3 µm) was used with a linear water/methanol gradient containing 0.1% formic acid. The instrument was operated in positive ESI mode, and 50 µL of sample was injected for each measurement.

## Author Contributions

Y.Y. proposed this project, designed all the experiments, performed all the experiments and data analysis, and wrote the manuscript. V.T.S. conducted the EPR experiments, simulated the data, helped Y.Y. analyze the data, and revised the manuscript. S.W. provided the ICP‐MS and HPLC‐MS measurements and analysis. M.M. acquired and shared the STEM images. M.P. supervised and guided the entire research project and manuscript writing.

## Conflicts of Interest

The authors declare no conflicts of interest.

## Supporting information




**Supporting File 1**: smll72966‐sup‐0001‐SuppMat.pdf.

**Supporting File 2**: smll72966‐sup‐0002‐VideoS1.mp4.

**Supporting File 3**: smll72966‐sup‐0003‐VideoS2.mp4.

**Supporting File 4**: smll72966‐sup‐0004‐VideoS3.mp4.

## Data Availability

The data that support the findings of this study are available from the corresponding author upon reasonable request.
