# Supplementary material for: Silver‐enhanced Photoresponsive g‐C3N4/Ag Janus Microrobots With Negative Photogravitaxis Efficient Antibiotic Degradation
Source: Small. 2026 Mar 11;22(24):e12272. doi: 10.1002/smll.202512272 (PMC13114499; doi:10.1002/smll.202512272)
Supplement: Supplementary file 1 — Supporting File 1: smll72966‐sup‐0001‐SuppMat.pdf. [file SMLL-22-e12272-s003.pdf]

Silver-enhanced Photoresponsive g-C_3N_4/Ag Janus Microrobots with Negative Photogravitaxis Efficient Antibiotic Degradation

Yunhuan Yuan,[1] Vinicius Tadeu Santana,[2] Stanisław Wacławek,[3] Michal Mazur,[4] Martin Pumera[1,5,6,7*]

[1]Future Energy and Innovation Laboratory, Central European Institute of Technology, Brno University of Technology, Purkynova 123, Brno, 61200, Czech Republic

[2]Central European Institute of Technology, Brno University of Technology, Purkynova 123, Brno, 61200, Czech Republic

[3] Institute for Nanomaterials, Advanced Technology and Innovation, Technical University of Liberec, Liberec 461 17, Czech Republic

[4] Department of Physical and Macromolecular Chemistry, Faculty of Science, Charles University, Hlavova 8, Prague 2 128 43, Czech Republic

[5]Department of Medical Research, China Medical University Hospital, China Medical University, No. 91 Hsueh-Shih Road, Taichung, TW-40402, Taiwan

[6]Advanced Nanorobots & Multiscale Robotics Laboratory, Faculty of Electrical Engineering and Computer Science, VSB – Technical University of Ostrava, 17. listopadu 2172/15, Ostrava, 70800, Czech Republic

[7]Department of Chemical and Biomolecular Engineering, Yonsei University, 50 Yonsei-ro, Seodaemun-gu, Seoul 03722, Korea

* Author for correspondence: martin.pumera@ceitec.vutbr.cz

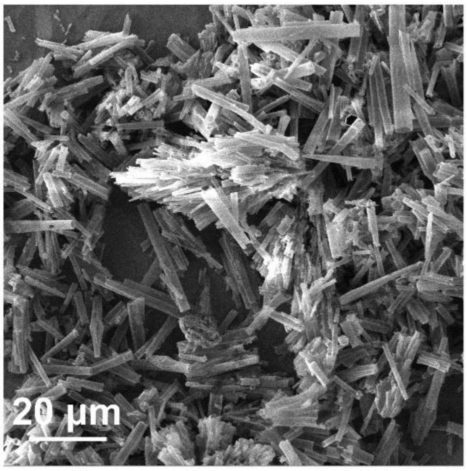

Figure S1. The SEM image of g-C_3N_4/Ag Janus microrobots.

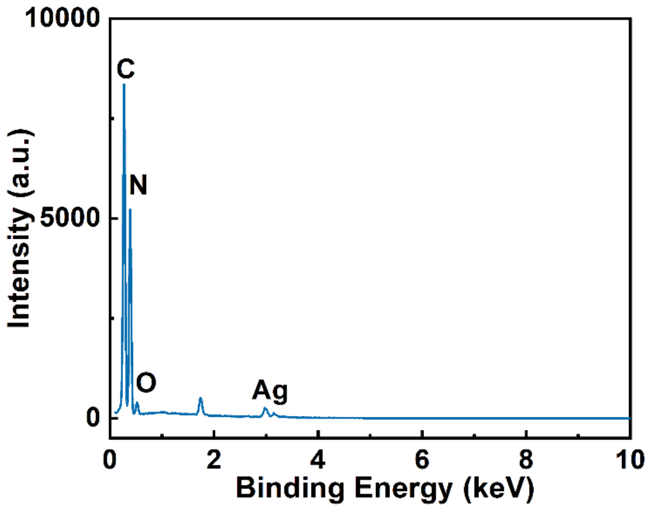

Figure S2. EDX spectrum of g-C_3N_4/Ag Janus microrobots.

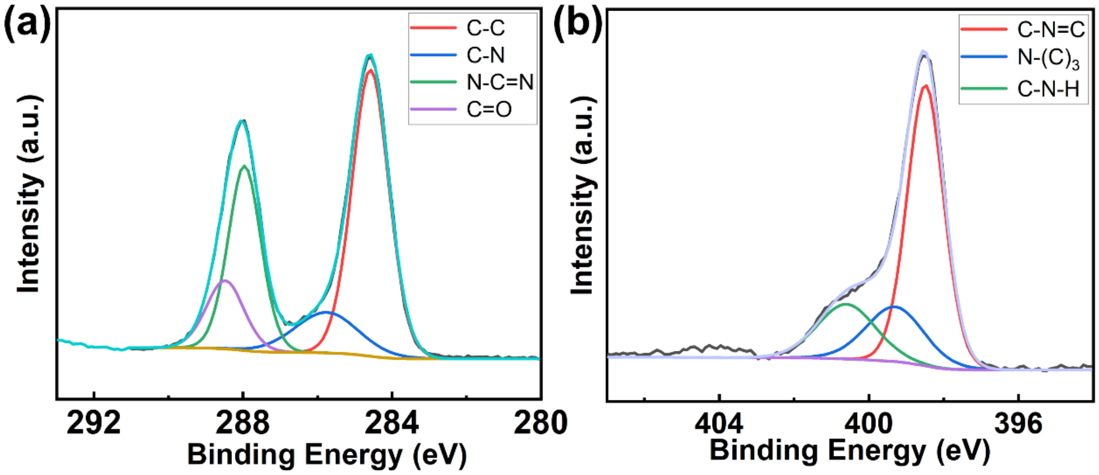

Figure S3. X-ray photoelectron spectroscopy (XPS) spectra of (a) C 1s and (b) N 1s.

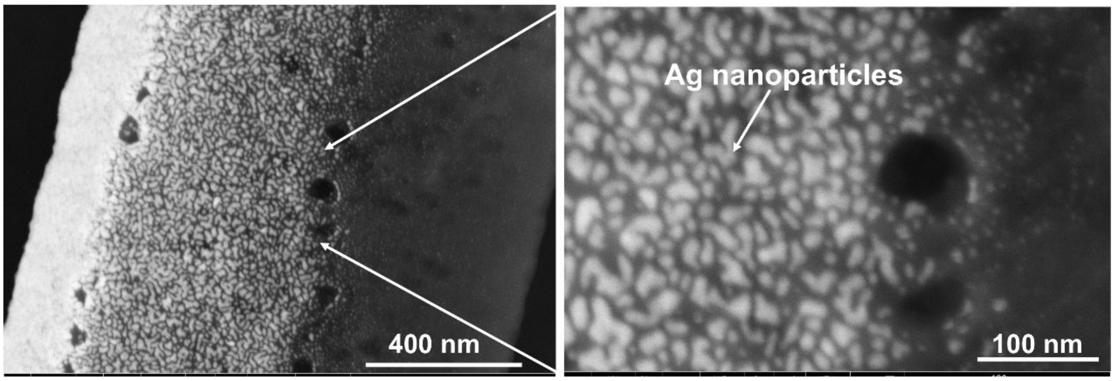

Figure S4. Enlarged SEM image of g-C_3N_4/Ag microrobots to show the surface morphology.

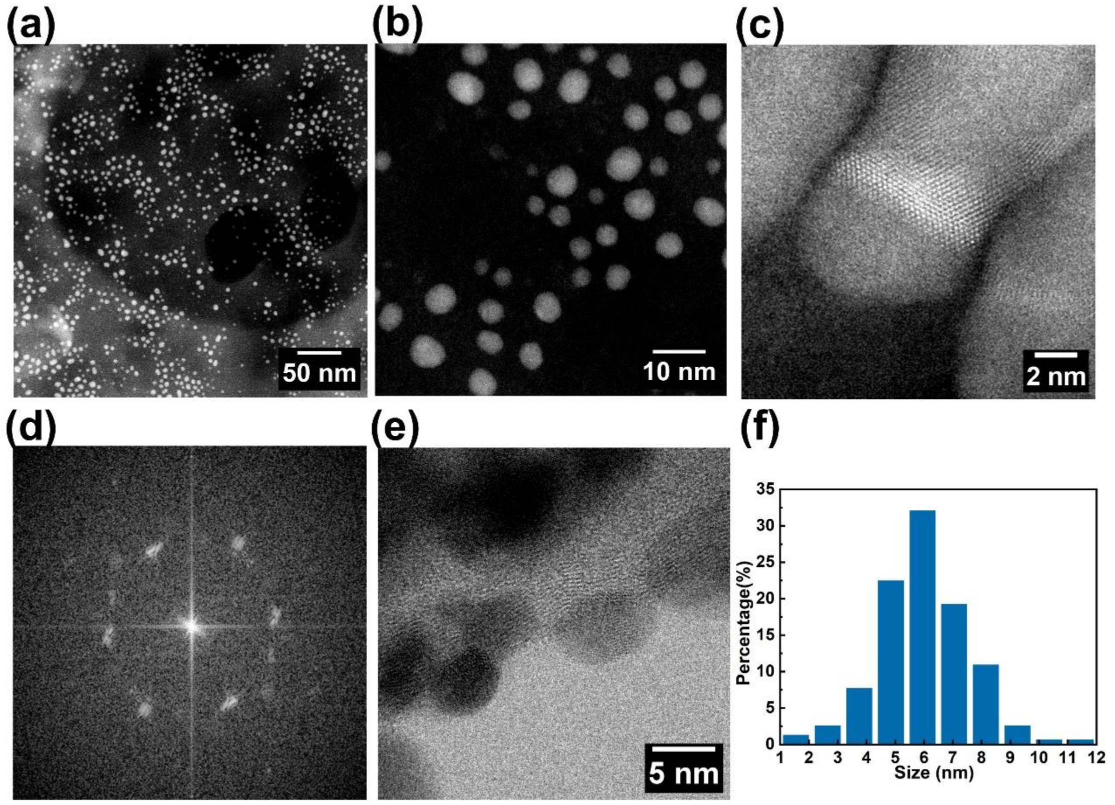

Figure S5. Scanning transmission electron microscopy (STEM) imaging of Ag nanoparticles on g-C_3N_4 microtubes. (a) STEM image showing the distribution of Ag nanoparticles on the g-C_3N_4 microtube. (b) High-magnification STEM image showing Ag nanoparticles with strong contrast on the g-C_3N_4 microtube. (c) High-magnification STEM image revealing clear lattice fringes of crystalline Ag nanoparticles and their interface with the g-C_3N_4 support. (d) Fast Fourier transform (FFT) pattern obtained from the STEM image in (c), confirming the crystalline nature of the Ag nanoparticles. (e) ABF-STEM image showing lattice fringes of crystalline Ag nanoparticles in direct physical contact with the g-C_3N_4 support, without obvious interfacial gaps. (f) Particle size distribution histogram of Ag nanoparticles, obtained from statistical analysis of more than 100 particles.

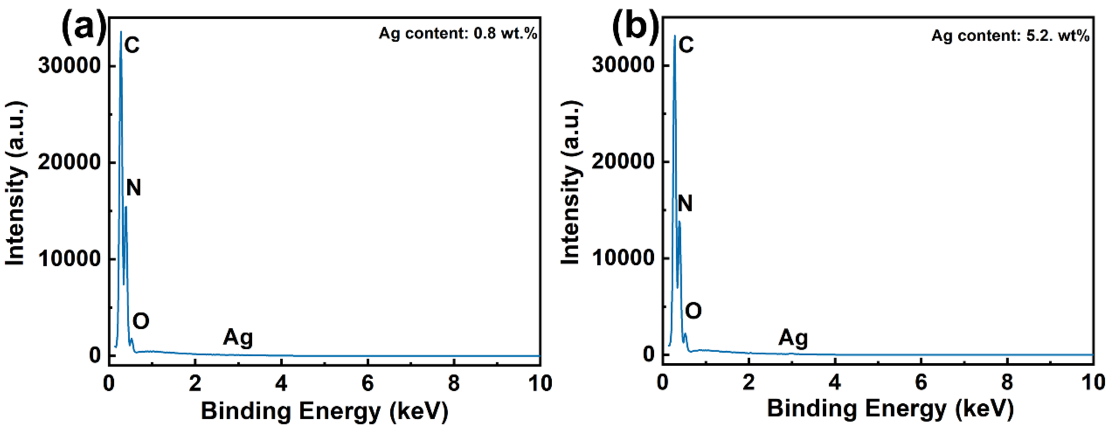

Figure S6. EDX spectra of two g-C_3N_4/Ag samples prepared from the same batch of Janus microrobots. (a) Sample A directly transferred onto the SEM stub with the uncoated side facing upward. (b) Sample B mechanically scraped off to expose the Ag-coated side.

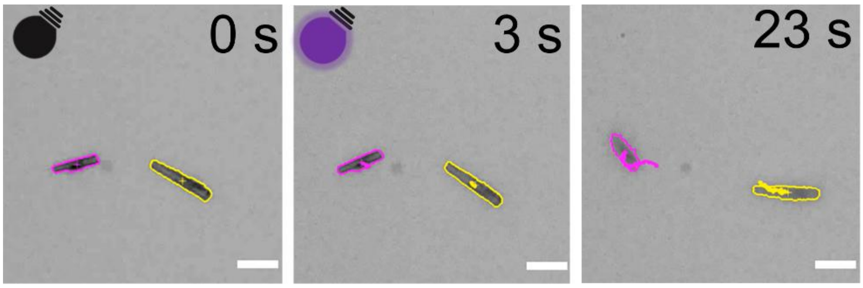

Figure S7. Motion trajectories of g-C_3N_4 in 0.2 wt% H_2O_2 solution under dark conditions (0–3 s) and UV illumination (3–23 s). Scale bars: 10 μm.

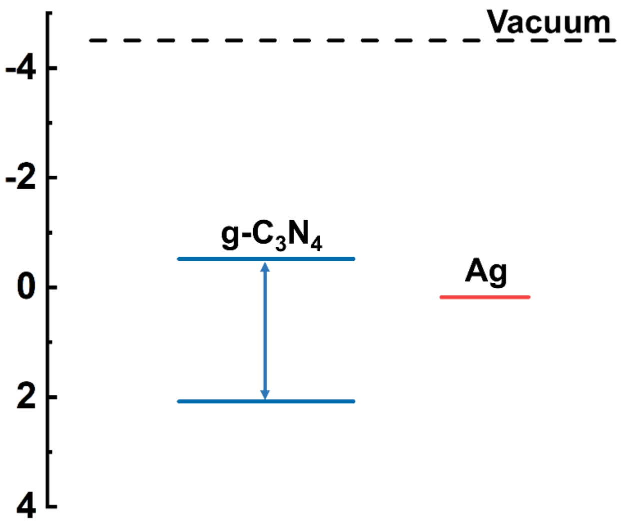

Figure S8. Conduction band (CB) and valence band (VB) positions of unmodified g-C_3N_4, along with the work function of Ag.

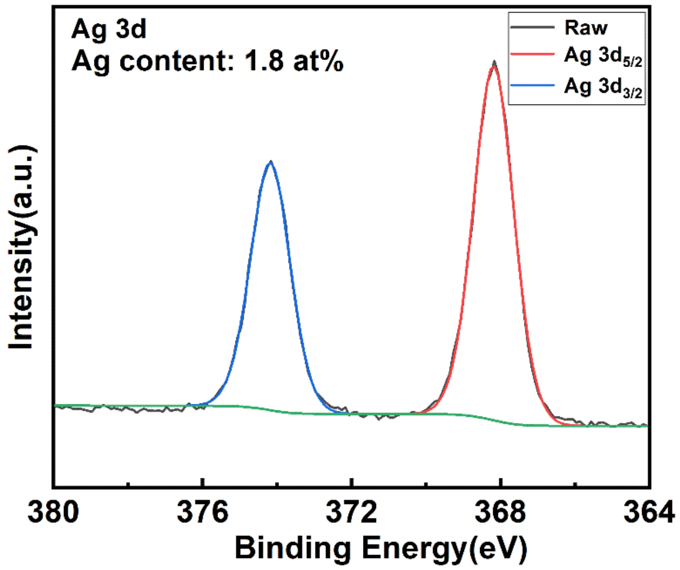

Figure S9. Ag 3d XPS spectrum of the non-Janus g-C_3N_4/Ag composite.

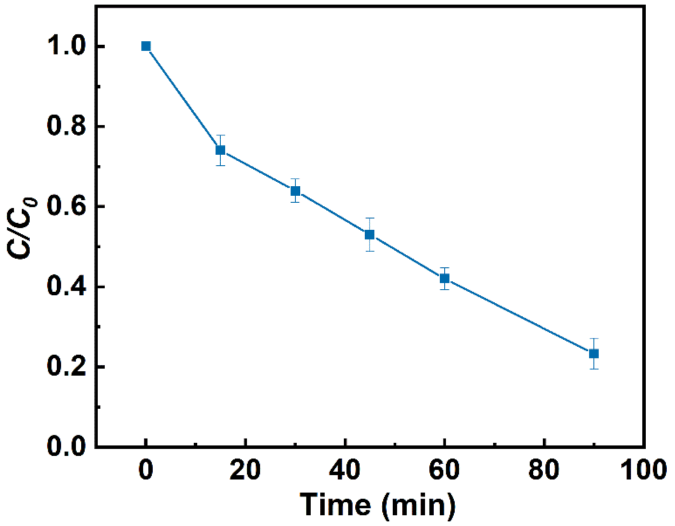

Figure S10. Photocatalytic degradation curves of tetracycline over time using the non-Janus g-C_3N_4/Ag composite under light irradiation in the presence of 0.2% H_2O_2 (N = 3).

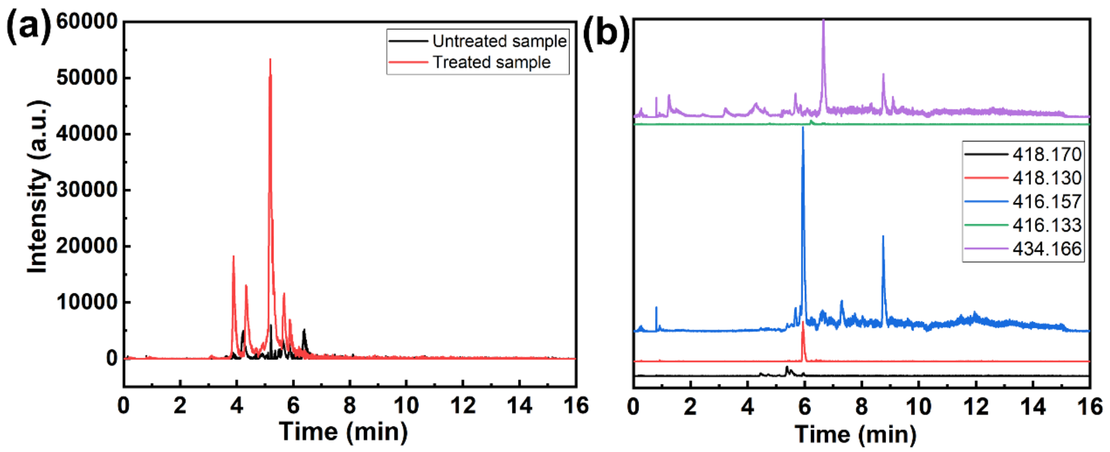

Figure S11. HPLC–MS analysis of tetracycline before and after photocatalytic treatment. (a) Total ion chromatograms (TIC) comparing the untreated tetracycline solution (black line) with the photocatalytically treated sample (red line). Multiple degradation product peaks appear at 3–5 minutes in the treated sample. (b) Extracted ion chromatograms (EICs) of degradation intermediates in the photocatalytically treated sample. Identified products include: oxidized form at m/z 418.130 (TCKOx), dehydrated form at m/z 416.133 (TCKdehyd), and hydroxylated derivative at m/z 434.166. Two additional unidentified intermediates are detected at m/z 418.170 and m/z 416.157. None of these species are detected in the untreated control sample. The detection of multiple transformation products with distinct m/z values indicates complex degradation pathways involving different chemical transformations.

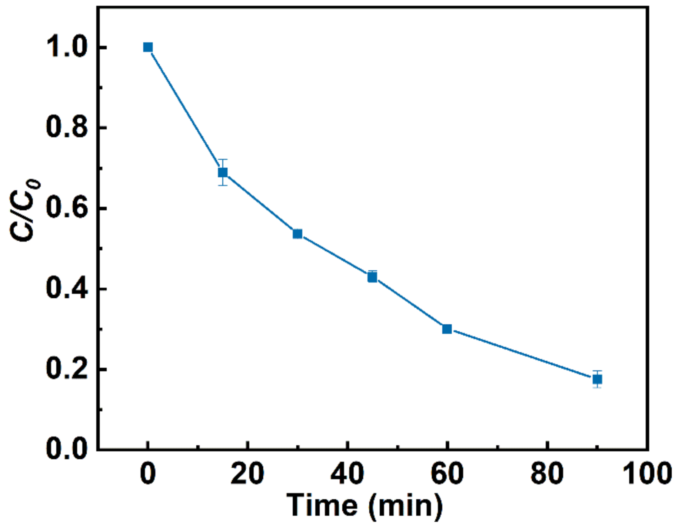

Figure S12. Photocatalytic degradation curves of tetracycline over time using g-C_3N_4/Ag Janus microrobots under light irradiation in the presence of 0.2% H_2O_2 in real wastewater (N = 3).

Table S1. EPR parameters used in the simulation of the spectra in **Figure 6b-d**.

			Hyperfine coupling constant (G)		
	Spin adducts	Diastereoisomers (%)	a_N	a_H^{β}	a_H^{γ}
Microrobots + Light + H_2O_2 + BMPO	BMPO/•OH	12	14.10	15.89	0.63
		49	14.17	12.81	0.68
	BMPO/•O_2^-	19	13.49	12.06	-
		20	13.49	9.75	-
Microrobots + Light + BMPO	BMPO/•OH	17	14.01	15.92	0.64
		60	14.10	12.79	0.68
	BMPO/•O_2^-	9	13.40	12.10	-
		14	13.40	9.40	-
Light + H_2O_2 + BMPO	BMPO/•OH	26	14.01	15.92	0.65
		61	14.10	12.79	0.69
	BMPO/•O_2^-	5	13.56	12.25	-
		8	13.56	9.51	-
